# Milk and Fresh Cheese Quality of Crossbred Cows Supplemented with Phytogenic Additives and Managed under Thermal Stress

**DOI:** 10.3390/ani13213402

**Published:** 2023-11-02

**Authors:** Abner Alves Mesquita, Poliana Carneiro Martins, Patrick Bezerra Fernandes, Leonardo Amorim de Oliveira, Paulo Victor Toledo Leão, João Antônio Gonçalves e Silva, João Vitor Teixeira da Cunha, Leandro Pereira Cappato, Ruthele Moraes do Carmo, Pedro Paulo Alves Pinheiro, Mariana Borges de Castro Dias, Edmar Soares Nicolau, Marco Antônio Pereira da Silva

**Affiliations:** 1 Instituto Federal de Educação, Ciência e Tecnologia Goiano, Rodovia Sul Goiana, Km 01, Zona Rural, Rio Verde 75901-970, Brazil; abnervet@gmail.com (A.A.M.); polianamartinsvet@hotmail.com (P.C.M.); leaonardoamorim@gmail.com (L.A.d.O.); paulovtbpv@gmail.com (P.V.T.L.); joao.antoniogs@hotmail.com (J.A.G.e.S.); texera07.jvt@gmail.com (J.V.T.d.C.); leandro.cappato@ifgoiano.edu.br (L.P.C.); pedropinheiro403@gmail.com (P.P.A.P.); maborges93.mb@gmail.com (M.B.d.C.D.); marco.antonio@ifgoiano.edu.br (M.A.P.d.S.); 2 Programa de Pós-Graduação em Ciência Animal, Universidade Federal de Goiás, Nova Veneza, Km 8, Goiânia 74690-900, Brazil; ruthele.carmo@italac.com.br (R.M.d.C.); rena@cpa.evz.ufg.br (E.S.N.)

**Keywords:** blood count, body temperature, milk product, shading, whey

## Abstract

**Simple Summary:**

This study focuses on the impact of environmental conditions and the use of phytogenic additives on dairy cattle performance and cheese production. The research was conducted in Brazil and considered the effects of shade and dietary additives on various physiological and production parameters. The results showed that providing shade to cows can help mitigate the effects of heat stress, although it did not significantly affect milk yield or quality. The use of a phytogenic additive did not yield substantial changes in the parameters studied, suggesting the potential adaptability of the animals to their environment. Cheese production was not significantly influenced by these factors, indicating a consistent yield and quality. This study highlights the need for further research to explore the benefits of phytogenic additives and their impact on animal welfare and product quality. In summary, while shade and dietary additives may offer some advantages, the adaptability of dairy cattle and specific environmental conditions should be considered when implementing strategies to enhance productivity and product quality in the dairy industry.

**Abstract:**

This investigation aimed to assess the physiological parameters and quality of milk and fresh cheeses produced by cows that were housed in paddocks, either with or without shade, and supplemented with a phytogenic additive. Sixteen crossbred cows were allocated in a 4 × 4 Latin square design, dividing them into paddocks with or without shade, and providing or not providing a phytogenic additive in their feed. This resulted in a total of four treatment groups and sixteen experimental plots, each containing four animals, over four periods of 21 days. Various parameters were examined, including haematology, rectal and skin temperature, respiratory rate, milk yield and composition, serum parameters, and cheese yield and quality. It is worth noting that the temperature and humidity, as measured by a black globe thermometer, did not display significant variations between the different environments and exhibited minimal fluctuations throughout the day. Additionally, the supplementation of the phytogenic additive led to a reduction in haematocrit levels (*p* = 0.011). Furthermore, the analysis showed that whey obtained from cheese production had a higher fat content when cows were without access to shade (*p* = 0.005). Notably, there was an interaction between factors in relation to the total dry extract content, which was lower when cows had access to shade and received the additive (*p* = 0.010). In summary, the provision of a phytogenic additive and the presence or absence of shade did not bring about significant changes in milk production and quality or in the yield and quality of fresh cheese.

## 1. Introduction

Dairy cattle perform best within a thermally neutral zone, while high or low temperatures lead to stress, restricting productive performance. These temperatures do not follow a single pattern, varying between the different species, ages, and physiological conditions of each animal [1].

For the animal to be in thermal equilibrium, its setting must be comfortable. Metabolic heat should be systemically lost to the environment without any kind of damage to the body [2].

Natural shading reduces direct solar radiation, avoiding the animals experiencing caloric stress and minimising productive losses. In the absence of natural shade, artificial shade can be used [3].

Cattle have the ability to identify shaded places that protect against solar radiation [4]; therefore, it is of paramount importance to use natural or artificial shade in order to reduce the stress caused by high temperatures, which consequently will lead to better productive performance of the animals. An understanding of the daily and seasonal changes in animal physiology allows for the better adoption of adjustments that will result in a greater comfort zone for the animals [5].

There are several ways to assess the response of the animal organism to physiological processes that indicate welfare in ruminants, and verification of the blood count is one of these [6,7,8]. Thermal stress triggers changes in behavioural reactions due to physiological changes in animals [9]. Haematological variables, as well as certain phenotypic characteristics, help characterise caloric stress in dairy cattle [5].

The sensitivity of dairy cows to thermal stress is quite evident, presenting responses such as a decrease in yield, reproduction, and welfare. High-yield animals subjected to thermal stress may present reduced food consumption; thus, their nutritional needs are not met, leading to a decrease in milk yield, milk solids, and the yield of derivatives [10], such as fresh cheeses.

In the future, the utilisation of phytogenic additives holds promise as a potential solution for mitigating the adverse impacts of heat stress. However, their viability hinges on ongoing research efforts aimed at assessing their suitability for this specific purpose. Presently, phytogenic additives are already integrated into livestock diets, with the primary objective of enhancing the quality of the end product. This approach strives to yield premium products free from any residues, thereby assuring consumers of excellent quality. Notably, these additives, encompassing plant extracts and their active components, offer the added benefit of being non-detrimental to animal health [11,12].

Studies are needed to evaluate the effects of heat stress on animals and on milk composition and the benefits of using phytogenic additives, in order to deliver technical and scientific information to producers and processing industries, aiming at the production of raw materials and quality derivatives.

Therefore, the objective of this study was to evaluate physiological parameters, milk quality, and fresh cheeses obtained from crossbred cows raised in an environment with or without shade and supplemented or not with phytogenic additives.

## 2. Materials and Methods

The project was approved by the Committee of Ethics in the use of animals of the Federal Institute of Goiás with protocol of approval number 7080110917.

The research was conducted at the Federal Institute of Goiás—Campus Rio Verde, located in Rio Verde, Goiás, Brazil (17°48′67″ S and 50°54′18″ W, with an elevation of 754 m). The experiment began in June 2018 and ended in September 2018. According to the Köppen classification, the region’s climate is classified as Aw, indicating a warm and humid climate with an annual precipitation range of 1500 to 1800 mm and an annual temperature range of 20 °C to 35 °C.

In this study, 16 crossbred cows of varying ages were used. Therefore, a specific calving order was not determined, as the herd had different ages. However, to maximise homogeneity within each treatment group, the cows were weighed and grouped in such a way that the average weight of each group was similar (466.5 ± 23.5 kg). A 4 × 4 Latin square design was adopted, with four treatments and four animals per treatment, and a 2 × 2 factor scheme, considering the environment in which the cows were raised (sun or shade) and the supply of phytogenic additives (yes or no). The experiment was conducted in four periods, each lasting 21 days, the first 14 days being used for the cows’ adaptation to their diets and the rearing environment, and the final 7 days for data collection.

When raised in the shade environment, the animals remained in *Brachiaria brizantha* paddocks, with access to the 80.0% black shading screen, with dimensions of 25 × 25 m, installed 4 m from the ground. When raised in the sunny environment, the animals remained in *Brachiaria brizantha* paddocks without access to the shading screen.

The phytogenic additive used (Biophytus-Prophytus Agroindustrial) was presented in powder form and was supplied mixed with the feed of the supplemented animals, at the amount of 2.26 g per day per animal, according to the manufacturer’s recommendation. To calculate this quantity, the average cow weight was multiplied by 1.9%, a reference value that indicates the consumption of dry matter per animal as a percentage of live weight [13], and divided by 100. Thus, the estimated dry matter consumption per animal (9.06 kg) was obtained, which was multiplied by 0.25, the recommended amount of additive per kg of dry matter consumed, reaching a value of 2.26.

According to the label, the additive consists of: copaíba oil (min. 40.00 g/kg), cashew oil (min. 240 g/kg), castor bean boil (min. 120 g/kg), calcareous seaweed, and silica.

All the cows had free access to water and pasture, according to the bromatological analysis presented in Table 1, and received corn silage ad libitum (Table 1), while the commercial feed, based on corn and soybean meal (Table 2), was supplied at the amount of 1 kg per animal. Samples of feed, pasture, and silage were sent to the ESALQ Lab Laboratory of the Zootechnical Department of the Luiz de Queiroz School of Agriculture (ESALQ) of the University of São Paulo (USP) for bromatological analysis in duplicate, by means of near infrared reflectance spectroscopy (NIRS). To obtain this information, the FOSS NIRSystem 5000 (Silver Spring, MD, USA) was used. The total digestible nutrients (TDN) were estimated using the equation recommended by Chandler [14].

Milking was performed twice a day at 07:00 and 15:00 h. Milk yield per animal was verified in the last two days of each 21-day period, that is, after 19 days of adaptation, with the aid of milk meters coupled with the milking equipment.

In the last two days of each period, samples of 40 mL of milk produced by the cows were collected in a bottle containing bronopol^®^, to be sent to the Milk Quality Laboratory (MQL), the Food Research Centre (FRC), and the Veterinary and Zootechnical School (VZS) of the Federal University of Goiás (FUG)—Câmpus Samambaia. 

The somatic cell count (SCC) (flow cytometry-ISO 13366-2/IDF 148-2, from 2006 [15]), protein, fat, lactose, urea nitrogen in milk (UNM), and casein contents (Fourier transform infrared (FTIR) spectroscopy-ISO 9622/IDF 141 from 15/SEP/2013 [16]), as well as degreased dry extract (DDE) and total dry extract (TDE) (Gravimetry-ISO 6731/IDF 21 from 2010 [17]) contents were evaluated. The calculation for degreased dry extract (DDE) was performed according to the method defined in Brazil in 2014 [15]. It involves subtracting the percentage of fat (% FAT) from the percentage of total dry extract (% TDE) [18]. The SCC results were transformed into a logarithmic scale to eliminate the effects of extreme values.

On the last day of each trial period, the milk produced was used for the manufacture of fresh cheeses, following the methodology described by Furtado [19], with adaptations. Cheeses of approximately 500.0 g each were manufactured, and the yield was calculated according to the formula: yield = (kg cheese/kg milk) × 100. The amount of milk (kg) required to produce 1 kg of cheese was calculated. According to Vieira and Lourenço Júnior [20], it takes 7 L of milk to produce 1 kg of fresh cheese. The serum removed during the process was also measured (kg), and three 40 mL samples were collected by treatment, in a bottle containing bronopol, for referral to UFG and performance of the same analyses previously applied to milk samples.

The cheeses produced at the end of each experimental period were evaluated in triplicate in terms of physical-chemical attributes, according to the methodology described by Brazil [21], including analyses of pH, acidity (given in percentage of lactic acid), moisture, protein, fat, TED, and ashes. The fat in the dry extract (FDE) was calculated according to the formula: FDE (%) = fat × total dry extract.

Ordinance 146 of 7 March 1996, which approves the Technical Regulations on the Identity and Quality of Dairy Products [22], was used as the basis for classifying the cheeses according to the average fat content in the dry extract, following the intervals: extra-fat (min. 60.0%); fat (between 45.0% and 59.9%); middle-fat (between 25.0% and 44.9%); lean (between 10.0% and 24.9%); and skimmed (under 10.0%).

On the 20th and 21st days of each period, at the time of milking in the morning and afternoon, the physiological parameters were evaluated, totalling six days of evaluation per period. The evaluated parameters were: the respiratory frequency per minute, counted by the number of movements of the flank during this time; the rectal temperature, measured by means of a digital clinical veterinary thermometer inserted in the rectum of the animals; and the skin temperature, recorded with the aid of an infrared digital thermometer, measured in different places of the body of the animal and calculated in accordance with the following formula: skin temperature (T) = 0.10 × T.head + 0.7 × T.back + 0.12 × T.cinnamon + 0.08 × T.udder [23].

On the last day of each period, a collection of 5.0 mL of blood per animal was obtained from the mammary vein, packed in a tube containing the anticoagulant heparin, and was sent to a private laboratory for clinical analysis, specifically for the evaluation of haemoglobin, haematocrit, medium corpuscular volume (MCV), medium corpuscular haemoglobin (MCH), medium corpuscular haemoglobin concentration (MCHC), neutrophils, eosinophils, lymphocytes, and monocytes. The CBC was performed in automatic equipment for counting blood cells (ABX VET PACK) using a specific reading card for cattle [24].

The maximum, minimum, and current temperature and relative humidity in the sun and shade paddocks (in the area covered by the shading screen) and in the milking parlour were checked before milking, performed at 07:00 and 15:00 h, daily, with the help of a thermohygrometer AK 28 new (AKSO^®^), with a 1.5 m cable sensor, storage of maximum and minimum temperature/humidity records, a temperature measurement range from −50 °C to 70 °C and a relative humidity measurement range up to 99%.

The temperature and relative humidity at the time of milking, i.e., of the moment, were also checked with a black globe thermometer. The standard black globe thermometer consists of a hollow sphere, with a diameter of around 0.15 m and a thickness of 0.5 mm, with the external part painted in matte black, and a temperature element inside, which can be a simple thermometer. For the present study, an adapted black globe thermometer was used, according to the manufacturing methodology of Souza et al. [25], who reported good performance in the functioning of their equipment.

For temperature and humidity, a fully causal design was used, considering the milking parlour environment, sun paddocks, and shade paddocks, as treatments, evaluated at two different times, namely, the morning and the afternoon.

Before conducting an analysis of variance, the data underwent a residual analysis (homogeneity of variances and normality tests). Subsequently, the data were subjected to an analysis of variance, where the means were compared using the Tukey test, all at a 5% probability level. R software (https://www.R-project.org/) was used to perform the analyses.

## 3. Results and Discussion

Table 3 shows the averages of the minimum, maximum, and relative temperatures and humidities at the time of measurement, verified in the sun, shade, and parlour environments with thermohygrometers, in addition to the black globe temperature and humidity recorded at the time of measurement. Brazil is a country with various types of climates, which in turn has a wide variation in temperature and humidity, and the predominant tropical climate in the Midwest expresses great thermal amplitude, as was observed.

The highest temperatures recorded with the thermohygrometer at the time of the measurements, throughout the day, correspond to the paddocks without shading (*p* = 0.022 in the morning and *p* < 0.001 in the afternoon). In environments with high temperatures associated with high solar incidence, it is ideal to provide shade to animals, which leads to a reduction in body heat and helps with thermoregulation. Shade is highly recommended in hot regions because it helps in body temperature regulation, favouring heat loss [26].

The shading in pastures creates possible places of rest, which favours rumination, and consequently, greater use of ingested food, with less wear and tear seen in animals that have not been exposed to the sun.

The milking parlour was the environment that maintained the lowest oscillation of maximum, minimum, and moment temperatures and humidities recorded with the thermohygrometer, both in the morning and afternoon (Table 3).

Although the day shifts were not compared statistically, it can be observed that within the same environment, the afternoon period always showed higher temperatures and lower relative moisture, recorded with either the thermometer or the black globe thermometer (Table 3). The absorption of solar radiation by the Earth’s surface occurs throughout the day, and at noon there is a solar incidence directed with greater intensity to regions of the tropics, where we are located. This implies the need to use natural shading/vegetation, and/or artificial shading with shadows, or sheds with ventilation, regardless of the time of year.

The temperatures and relative moisture of the moment, in the morning and afternoon periods, recorded with the black globe thermometer, showed less variation when compared to the same measurements recorded with the thermometers (Table 3). Also, they did not present any statistical difference between the compared environments (*p* = 0.406 and 0.699 for temperature and humidity in the morning, respectively, and *p* = 0.360 and *p* = 0.547 for temperature and humidity in the afternoon, respectively) different from the averages recorded with the thermometer, which were significant in their majority.

A black globe thermometer is efficient in isolating the average radiant temperature from other thermal factors of the environment [25], which may explain the smallest variation in the measurements obtained through this one.

The measurements of the black globe temperature cannot be applied as a definitive parameter for environmental thermal comfort evaluations, due to the physiological differences between the animals, but it is widely used in monitoring for the environmental thermal comfort analysis of the animals, due to its characteristics of gathering, in a single result, many climatological variables, such as relative air humidity, temperature, wind speed, and solar radiation [26].

Regarding the complete blood count, there was no effect of the treatments (*p* > 0.05) for the following variables: red blood cells, haemoglobin, MCV, MHC, MCHC, neutrophils, eosinophils, lymphocytes, and monocytes. The supplementation with phytogenic additives reduced the haematocrit value (*p* = 0.011), as shown in Table 4.

It was expected that access to shade would influence the blood parameters of the animals, as a consequence of thermal stress alleviation. However, only the phytogenic additive caused a change in haematocrit.

Studies developed by Gabbi et al. [27] pointed out that the use of plant extracts as an additive for dairy heifers increased the number of red blood cells, leukocytes, lymphocytes, and monocytes, probably preventing the oxidation of these cells.

Belic et al. [28] described a reduction in the number of RBC haematocrit and haemoglobin concentrations in dairy cows and attributed the changes to signs of hyperhydration, indicating active evaporation for body cooling, which did not occur in this study.

Paudel et al. [29] did not observe an effect of different frequencies of cold-water bathing, in dairy cows raised under high temperatures, on blood parameters such as haemoglobin, haematocrit, sodium, potassium, chloride, and bicarbonate levels. The same was reported in the work of Dalcin et al. [30], who concluded that thermal stress did not change the haematocrit and haemoglobin concentration of pure or mixed Holstein dairy cows.

On the other hand, Mazzullo et al. [31] observed that dairy cows raised at higher temperatures had reduced haemoglobin concentrations and red blood cell counts, decreased platelet percentage, and increased lymphocyte percentage as a physiological response to the imposed environmental challenge, showing the animals’ ability to adapt to thermal stress. The respiratory rate, rectal temperature, and skin temperature of the cows were not altered by the treatments (*p* > 0.05), neither in the morning nor the afternoon (Table 5).

Measuring the body temperature is important because, together with the measurements of environmental temperature and humidity with the black globe thermometer and dry bulb, it allows the determination of the relationships between the environmental climate, body temperature, and milk yield of the animals [32].

All variables showed an increase in values in the afternoon period, probably due to the variation in environmental temperature that normally occurs throughout the day, showing the importance of providing access to shade, mainly in the afternoon. In a study performed with Dutch cows subjected to different levels of artificial shade protection, it was found that 73.3% of the animals spent more time under shade during the hottest hours of the day [4].

The shading of pastures through afforestation is efficient in improving the animals’ thermal comfort, which results in a lower respiratory rate, a reduced body surface temperature, and lower levels of sweating, leading to a reduction in energy expenditure for homeostasis maintenance [33].

Silva et al. [34] evaluated the exposure of animals to the sun at different times of the day and observed respiratory rate values similar to the results found in this study. According to Silanikove [35], the following respiratory frequencies indicate the probability of a bovine suffering thermal stress: low—40 to 60 movements per minute; medium—60 to 80 movements per minute; high—80 to 120 movements per minute; severe—above 150 movements per minute. Taking into consideration these parameters, the results obtained in this study showed that the animals were not under thermal stress, because their respiratory movements were below 60 movements per minute.

The rectal temperature of dairy cattle has a mean value of 38.5 °C [36], and the results expressed by the animals raised in both environments, both receiving and not receiving the phytogenic additive, regardless of the period of the day, are close to this value, with 38.29 °C being the highest mean obtained. The rectal temperature is a physiological parameter that indicates the amount of heat accumulated by the animals during a given period, presenting higher values at the end of the day and at times of greater solar radiation [37].

In a study developed by Silva [34], the solar incidence at certain times resulted in higher rectal temperatures of the animals, according to the authors, by influencing an increase in endogenous heat production. The results of Arcaro Junior et al. [38], in turn, showed that animals located in areas with artificial shading showed increased milk yield and lower body and rectal temperatures.

Furthermore, the phytogenic additive used, based on copaíba, castor bean, and cashew oils, did not cause significant changes in the parameters evaluated in Table 5. According to the manufacturer, the action of the additive is to maximise the productive potential of the animals by reducing body wear through better physiological use [39], and this wear was not observed in the present study when considering the physiological parameters described above.

Gabbi et al. [27], in turn, when testing a commercial mixture of essential oils, flavonoids, and mucilage as a phytogenic additive in the feeding of Jersey dairy heifers, found that animals that received the additive in their feed had lower heart rates, compared to the group that did not receive the additive.

The yield, centesimal composition, and SCC of milk were not affected (*p* > 0.05) either by the breeding environment or by the use of phytogenic additives (Table 6).

Dairy cows, especially those with a high milk yield, have accelerated metabolisms and a high rate of metabolic heat production. When suffering from thermal stress, they reduce their dry matter consumption, which leads to a reduced milk yield [40].

However, in this study, there was no change in milk yield, suggesting that the stress caused was not sufficient to cause this change, or that there was adaptation of the animals to the environment.

Silva et al. [34] reported a significant reduction in the milk yields of cows subjected to direct sunlight from 10:00 a.m. to 12:00 p.m., when compared to cows with access to shading at that time. Almeida et al. [41] did not observe significant changes in milk quality or chemical composition of cows exposed to an evaporative cooling system, when compared to a group of animals that were not submitted to the cooling system. On the other hand, the group of animals submitted to 30 min air conditioning in the waiting corral showed a higher frequency of access to the trough and feeder, indicating lower levels of stress, which were associated with improved welfare and a consequent increase in milk yield.

When there is thermal stress, milk protein is negatively affected, with a decrease in casein, calcium ion, and phosphorus levels, probably due to lower fodder intake [42]; this change was not observed in the present study. Fat levels decrease when cows are exposed to caloric stress that is classified as severe. Thermal stress may increase the susceptibility of the animal to infections [43], which could influence the values of SCC, a result not observed in this study.

As there were no significant changes in milk yield and composition, it is possible that the thermal stress experienced by the animals was not sufficient to promote a drop in yield performance and milk quality, either because of the time of year in which the experiment was conducted or due to the possibility that the cattle had already adapted to the environment in which they were raised.

Urea is one of the main products of nitrogen metabolism in farm animals, and much of the urea produced is normally excreted in urine, while the rest is integrated into the blood and transferred to the milk [44].

Since the additive is intended to enhance animal performance, it was anticipated that alterations in digestive processes due to supplementation might lead to elevated ammonia levels in the rumen and, consequently, higher urea nitrogen content in milk.

Urea is a primary means of nitrogen compound excretion in animals. In dietary terms, the quantity of protein directly influences urea levels, with protein-rich diets increasing them and a reduced protein intake having the opposite effect. Diets with low energy levels or low protein quality favour an increase in urea amounts because of protein catabolism [45].

Table 7 shows the percentage of whey obtained in the manufacturing of cheeses, as well as the analysis of its centesimal composition and SCC. There was no effect of environment or additive (*p* > 0.05) on whey yield, protein percentages, lactose, DDE, UNM, casein, or SCC.

Whey is a by-product of cheese production and is considered a raw material of high nutritional value and low cost, presenting several possibilities for human nutrition, in addition to being made of proteins with high biological value [46].

Between 80.0% and 90.0% of the total volume of milk used in the manufacture of cheese returns as whey, which contains 55.0% of all the nutrients in milk [47]. The whey consists of 93.0% water, 5.0% lactose, 0.7–0.9% protein, 0.3–0.5% fat, 0.2% lactic acid, and vitamins [48]. The recovery of whey components may generate extra gains for the dairy industry, which may increase profitability due to the better use of this co-product.

The percentage of fat in the whey remaining after the production of the cheeses was higher for cows raised in the paddocks in the sun (*p* = 0.005) (Table 7). This shows that the animals that remained exclusively in the sun did not have impaired residual fat levels in their milk, suggesting the adaptation of the herds to the climate into which they were inserted, which is beneficial to the dairy production chain.

There was an interaction effect of environment x additive on TSE content, which was lower when cows had access to shade and received the additive (*p* = 0.005), contrary to what was expected. TDE is very important in the production of whey derivatives and other foods that use it in their composition, such as cakes, meat products, such as mortadella, and derivatives, such as ricotta and dairy beverages, whose functional and nutritional characteristics reflect the quality of the whey [49,50]. The TSE values found in this study are generally below those described in the literature, since contents between 11.0% and 13.0% are mentioned [51,52].

Milk demands a considerable amount of metabolic work in order to be produced. In dairy cattle, 450 L of blood must pass through the mammary gland to produce one litre of milk. The quantity and quality of milk produced varies according to breed or individual characteristics, and the main constituents found include: water, 87.3%; lipids, 3.7%; lactose, 4.8%; casein, 2.9%; globulins, 0.6%; and ash, 0.72% [44]. These constituents are important for the dairy production chain; fat, for example, gives rise to buttermilk, one of the products with the highest added value; casein is the key component in the production of all types of existing cheeses; and globulins and lactose add value to the whey used in the production of milk beverages and for the production of “whey proteins”, used by people following a healthy lifestyle.

The thermal stress promoted by the shade-free environment and the supply of phytogenic additives were not able to alter the yield of fresh cheeses (*p* > 0.05) (Table 8).

The number of total solids, i.e., the TDE of milk, directly influences the yield of cheese, which means that higher levels of lactose, and especially of fat and protein (namely casein), determine a better yield [53].

Thermal stress was expected to reduce the yield of the cheeses indirectly, by altering the composition of the milk. However, since the milk composition did not change (Table 6), the cheese yield did not change either.

According to Summer et al. [54], the climatic conditions under which cows are raised can also affect the pH and titratable acidity of milk, which are essential for the cheese manufacturing process, both for its yield and quality. Thus, cows subjected to thermal stress result in the production of cheeses with lower yields.

The yield of cheeses is an important variable, since it is related to the profit obtained by the industry. Although there was no significant difference found, the product of cows that received shade and additives can be considered differentiated by the conditions to which the animals were exposed and can therefore serve as a marketing strategy for consumers who care about animal welfare.

Table 9 presents the results regarding the physical–chemical attributes of cheeses. The variables of pH, acidity, and percentages of moisture, protein, fat, TDE, FTD, and ash were not influenced by the treatments (*p* > 0.05).

Acidity in dairy products is an important parameter for evaluating the quality of the raw material and also the type and quality of processing/storage that this product has received after manufacture. According to Order in Council 146 [18], all the cheeses produced fall within the classification of full-fat cheese, because they have a fat content of more than 45.0% and less than 59.9%. Fat is one of the most important components of cheese, and its function is to give colour to the rind of mature cheeses, as well as to give the cheese a pleasant taste. Structurally, it is responsible for the softness and lightness that are characteristic of fresh cheese.

The use of phytogenic additives, as well as the creation of better thermal comfort due to shading, were expected to result in a higher milk yield and the differentiated quality of the milk constituents, which would, in turn, result in a final product, i.e., cheese, of better quality. Such a differentiation was not found, which may be attributed to factors including the adaptability of the animals or the experimental period.

Additives in animal feed are a reality in the yield chain, having many diverse functions: improving immunity, feed conversion, and weight gain, for example. The greater use of natural additives, which can replace antibiotics and other artificial products, demands specific studies that evaluate all the physiology and benefits to the yield and welfare of the animals that receive them, in addition to the quality of the final product that reaches the consumer.

## 4. Conclusions

This study underscores the importance of providing shade in pastures to mitigate thermal stress in dairy cattle, significantly enhancing animal comfort and well-being. Surprisingly, the introduction of a phytogenic additive did not demonstrate substantial impacts on the studied parameters, indicating the need for further research to understand its effectiveness under various conditions.

The stability in milk production and composition across different environmental settings suggests the potential adaptation of the animals to their environment, or that the thermal stress levels were not severe enough to significantly affect these aspects of milk production. These findings are crucial for both the dairy industry and consumers seeking high-quality dairy products while ensuring the well-being of the animals involved in the production process.

## Figures and Tables

**Table 1:** Bromatological analysis of forage and silage supplied to animals during the experimental period.

Composition Analysis (Expressed in g/kg of DM)	*Brachiaria brizantha*	Maize Silage
Dry matter (%)	45.40	27.80
Crude protein	16.60	9.20
Acid detergent fibre	34.60	27.30
Neutral detergent fibre-free ash	59.00	Nc
Neutral detergent fibre	63.20	45.50
Starch	0.20	30.50
Ethereal extract	4.60	3.60
Mineral matter	12.70	4.70
Lignin	3.20	3.30
Calcium	0.53	0.19
Phosphorus	0.44	0.21
Potassium	2.72	1.08
Magnesium	0.32	0.13
Sulphur	0.29	0.12
Chloride	0.90	0.34
Total digestible nutrients (%)	60.00	70.00
Total fatty acids	2.11	2.33
C18:1-Oleic (%, AGT)	17.07	18.06
C18:2-Linoleic (%, AGT)	26.52	49.25
C18:3-Linolenic (%, AGT)	1.38	8.29

AGT—total fatty acids (fatty acid analyses were also obtained using NIRS).

**Table 2:** Feed supplied to the animals during the experimental period.

Guaranteed Levels per kg of Feed (Manufacturer’s Information)	Quantity
Maximum humidity (g)	120.00
Crude protein (minimum) (g)	220.00
Ethereal extract (minimum) (g)	25.00
Fibrous matter (maximum) (g)	100.00
Ash (maximum) (g)	80.00
Calcium (minimum) (mg)	5500.00
Calcium (maximum) (g)	13.00
Phosphorus (minimum) (mg)	3500.00
Acid detergent fibre (g)	120.00
Total digestible nutrients (minimum) (g)	740.00
Vitamin A (minimum) UI	9000.00
Vitamin D3 (minimum) UI	1900.00
Vitamin E (minimum) UI	19.00
Biotin (minimum) (mg)	0.16
Cobalt (minimum) (mg)	0.20
Copper (minimum) (mg)	13.00
Sulphur (minimum) (mg)	3320.00
Iron (minimum) (mg)	21.00
Iodine (minimum) (mg)	0.50
Magnesium (minimum) (mg)	1970.00
Manganese (minimum) (mg)	25.00
Selenium (minimum) (mg)	0.26
Sodium (minimum) (mg)	2720.00
Zinc (minimum) (mg)	65.00
Antioxidant BHT (minimum) (mg)	21.00
Yeast (*Saccharomyces cerevisiae*) (minimum) (ufc/g)	460.00
Monensin (minimum) (mg)	24.00
**Composition analysed (results expressed in 100.0% of DM)**	
Dry matter (%)	87.10
Humidity (%)	12.90
Crude protein (%)	24.50
Soluble protein (%, CP)	20.00
Fibre in acid detergent (%)	11.40
Fibre in neutral detergent (%)	17.20
Starch (%)	37.20
Ether extract (%)	4.00
Ash (%)	6.50
Non-fibrous carbohydrates (%)	47.80
Total digestible nutrients (%)	80.00

CP—crude protein; BHT—butylated hydroxytoluene.

**Table 3:** Temperature and humidity recorded during the experimental period.

Time	Ambient	Temperature (°C)			Relative Humidity (%)			Black Globe	
		Mom.	Max.	Min.	Mom.	Max.	Min.	T (°C)	RH (%)
Morning	Room	17.8 b	29.5 b	14.9 a	57.3 ab	64.2 b	20.6 a	20.4 a	62.4 a
	Sun	21.5 a	41.5 a	10.9 b	46.1 b	80.7 a	21.7 a	21.3 a	60.7 a
	Shade	18.3 ab	31.3 b	12.8 b	61.5 a	78.1 a	17.3 a	23.4 a	59.0 a
*p* value		0.022	<0.001	<0.001	0.035	<0.001	0.147	0.406	0.669
CV (%)		22.18	13.31	21.82	34.42	16.54	35.67	31.72	19.61
Afternoon	Room	27.8 b	29.2 c	17.4 a	30.2 a	61.9 ab	21.6 a	28.5 a	41.5 a
	Sun	36.4 a	40.6 a	19.8 a	23.1 ab	49.8 b	21.5 a	29.8 a	37.0 a
	Shade	29.3 b	33.0 b	17.4 a	18.6 b	69.0 a	16.8 b	30.1 a	37.7 a
*p* value		<0.001	<0.001	0.123	0.003	0.005	0.012	0.360	0.547
CV (%)		13.67	13.20	24.78	34.86	30.88	28.73	11.80	34.89

T °C—temperature; RH—relative humidity; Mom.—moment; Max.—maximum; Min.—minimum; CV—coefficient of variation. Means followed by the same lowercase letters in the columns do not differ from each other at a 5% significance level.

**Table 4:** Complete blood count of mixed cows submitted to different breeding environments and fed with phytogenic additives.

Variable	Ambient	Additive		Average	*p* Value			CV (%)
		Yes	No		Ambient	Additive	Ambient × Additive	
Red blood cell (mm^3^)	Sun	5.88 Aa	6.04 Aa	5.96 a	0.169	0.368	0.669	3.89
	Shade	5.75 Aa	5.81 Aa	5.78 a				
	Average	5.82 A	5.93 A	-				
Haemoglobin (g em 100 mL)	Sun	9.24 Bb	10.17 Aa	9.71 a	0.275	0.098	0.101	4.98
	Shade	9.42 Aa	9.42 Ab	9.42 a				
	Average	9.33 B	9.80 A	-				
Haematocrit (%)	Sun	28.10 Aa	30.00 Aa	29.05 a	0.205	0.011	0.126	2.44
	Shade	28.23 Aa	28.87 Aa	28.55 a				
	Average	28.16 B	29.43 A	-				
MCV (µ^3^)	Sun	47.75 Aa	49.54 Aa	48.64 a	0.907	0.099	0.289	2.37
	Shade	48.49 Aa	48.94 Aa	48.71 a				
	Average	48.12 B	49.24 A	-				
MHC (pg)	Sun	15.97 Aa	16.59 Aa	16.28 a	0.374	0.138	0.566	6.84
	Shade	16.17 Aa	17.48 Aa	16.82 a				
	Average	16.07 A	17.03 A	-				
MCHC (%)	Sun	33.00 Aa	33.00 Aa	33.00 a	0.355	0.355	0.355	0.38
	Shade	33.00 Aa	33.12 Aa	33.06 a				
	Average	33.00 A	33.06 A	-				
Neutrophils (%)	Sun	41.79 Aa	40.06 Aa	40.92 a	0.463	0.962	0.209	6.16
	Shade	41.00 Aa	42.85 Aa	41.92 a				
	Average	41.39 A	41.45 A	-				
Eosinophils (%)	Sun	5.06 Aa	5.56 Aa	5.31 a	0.135	0.370	0.618	20.26
	Shade	5.18 Aa	3.73 Aa	4.45 a				
	Average	5.12 A	4.64 A	-				
Lymphocytes (%)	Sun	51.25 Aa	52.25 Aa	51.75 aa	0.370	0.926	0.307	3.82
	Shade	51.39 Aa	50.20 Aa	50.80 aa				
	Average	51.32 A	51.22 A	-				
Monocytes (%)	Sun	1.89 Aa	2.12 Aa	2.01 a	0.133	0.398	0.803	30.61
	Shade	2.41 Aa	2.83 Aa	2.62 a				
	Average	2.15 A	2.47 A	-				

MCV—mean corpuscular volume; MHC—mean corpuscular haemoglobin; MCHC—mean corpuscular haemoglobin concentration; pg—per gram; CV—coefficient of variation. Averages followed by distinct uppercase letters on the line and lowercase letters on the column differ statistically (*p* > 0.05).

**Table 5:** Respiratory frequency, rectal temperature, and skin temperature of mixed cows submitted to different rearing environments and fed with phytogenic additive.

Variable	Moment	Ambient	Additive		Average	*p* Value			CV (%)
			Yes	No		Ambient	Additive	Ambient × Additive	
Respiratory frequency (movements /min)	Morning	Sun	36.93 Aa	39.37 Aa	38.15 a	0.388	0.359	0.858	16.46
		Shade	33.56 Aa	37.12 Aa	35.34 a				
		Average	35.25 A	38.25 A					
	Afternoon	Sun	42.93 Aa	44.43 Aa	43.68 a	0.688	0.315	0.809	5.12
		Shade	42.75 Aa	43.68 Aa	43.21 a				
		Average	42.84 A	44.06 A					
Rectal temperature (°C)	Morning	Sun	37.72 Aa	37.73 Aa	37.73 a	0.265	0.271	0.287	1.46
		Shade	37.74 Aa	38.40 Aa	38.07 a				
		Average	37.73 A	38.07 A					
	Afternoon	Sun	38.43 Aa	38.20 Aa	38.31 a	0.631	0.867	0.117	0.75
		Shade	38.10 Aa	38.39 Aa	38.24 a				
		Average	38.27 A	38.29 A					
Skin temperature (°C)	Morning	Sun	28.79 Aa	29.79 Aa	29.29 a	0.711	0.650	0.137	3.11
		Shade	29.39 Aa	28.83 Aa	29.11 a				
		Average	29.09 A	29.31 A					
	Afternoon	Sun	30.68 Aa	31.18 Aa	30.93 a	0.108	0.620	0.215	1.67
		Shade	31.53 Aa	31.31 Aa	31.42 a				
		Average	31.10 Aa	31.24 A					

CV—coefficient of variation. Averages followed by distinct uppercase letters in the line and lowercase letters in the column differ statistically (*p* > 0.05).

**Table 6:** Yield, centesimal composition, and SCC of milk from mixed cows fed with a phytogenic additive.

Variable	Ambient	Additive		Average	*p* Value			CV (%)
		Yes	No		Ambient	Additive	Ambient × Additive	
Yield (L)	Sun	9.84 Aa	10.52 Aa	10.18 a	0.750	0.533	0.917	13.15
	Shade	9.62 Aa	10.15 Aa	9.89 a				
	Average	9.73 A	10.34 A					
Protein (%)	Sun	3.58 Aa	3.63 Aa	3.60 a	0.396	0.603	0.713	3.44
	Shade	3.54 Aa	3.55 Aa	3.55 a				
	Average	3.56 A	3.59 A					
Fat (%)	Sun	3.68 Aa	3.78 Aa	3.73 a	0.167	0.895	0.265	3.99
	Shade	3.65 Aa	3.57 Aa	3.61				
	Average	3.67 A	3.68 A					
Lactose (%)	Sun	4.34 Aa	4.36 Aa	4.35 a	0.186	0.694	0.859	4.10
	Shade	4.19 Aa	4.25 Aa	4.22 a				
	Average	4.27 A	4.30 A					
TDE (%)	Sun	13.42 Aa	13.21 Aa	13.31 a	0.482	0.843	0.467	5.60
	Shade	12.85 Aa	13.22 Aa	13.03 a				
	Average	13.13 A	13.21 A					
DDE (%)	Sun	8.93 Aa	9.06 Aa	8.99 a	0.231	0.126	0.637	2.14
	Shade	8.76 Aa	8.98 Aa	8.87 a				
	Average	8.85 A	9.02 A					
UNM (%)	Sun	12.33 Aa	10.94 Aa	11.63 a	0.727	0.071	0.487	8.23
	Shade	11.80 Aa	11.12 Aa	11.46 a				
	Average	12.97 A	11.03 A					
Casein (%)	Sun	2.81 Aa	2.88 Aa	2.84 a	0.721	0.076	0.513	3.30
	Shade	2.76 Aa	2.89 Aa	2.83 a				
	Average	2.79 A	2.89 A					
SCC (×1000 SC per mL)	Sun	5.20 Aa	5.62 Aa	5.41 a	0.378	0.499	0.123	13.75
	Shade	6.26 Aa	5.29 Aa	5.78 a				
	Average	5.73 A	5.45 A					

TDE—total dry extract; DDE—degreased dry extract; SCC—somatic cell count; UNM—urea nitrogen in milk; CV—coefficient of variation. Averages followed by distinct uppercase letters in the line and lowercase letters in the column differ statistically (*p* > 0.05).

**Table 7:** Yield, centesimal composition, and CCS of whey used in the manufacture of fresh cheeses.

Variable	Ambient	Additive		Average	*p* Value			CV (%)
		Yes	No		Ambient	Additive	Ambient × Additive	
Yield whey (%)	Sun	73.96 Aa	76.03 Aa	74.99 a	0.104	0.160	0.240	1.88
	Shade	76.25 Aa	76.46 Aa	76.36 a				
	Average	75.11 A	76.25 A	-				
Protein (%)	Sun	1.16 Aa	1.10 Aa	1.13 a	0.471	0.831	0.445	8.76
	Shade	1.15 Aa	1.18 Aa	1.17 a				
	Average	1.15 A	1.14 A	-				
Fat (%)	Sun	0.59 Aa	0.60 Aa	0.59 a	0.005	0.129	0.266	3.00
	Shade	0.43 Aa	0.48 Aa	0.45 b				
	Average	0.51 A	0.54 A	-				
Lactose (%)	Sun	4.78 Aa	4.72 Aa	4.75 a	0.098	0.321	0.127	3.42
	Shade	4.48 Aa	4.71 Aa	4.59 a				
	Average	4.63 A	4.71 A	-				
TDE (%)	Sun	7.35 Aa	7.18 Aa	7.26 a	0.387	0.183	0.010	2.30
	Shade	6.97 Bb	7.40 Aa	7.19 a				
	Average	7.16 A	7.29 A	-				
DDE (%)	Sun	6.68 Aa	6.57 Aa	6.63 a	0.311	0.472	0.085	2.47
	Shade	6.42 Aa	6.65 Aa	6.54 a				
	Average	6.55 A	6.61 A	-				
UNM (%)	Sun	10.13 Aa	9.45 Aa	9.79 a	0.811	0.759	0.363	12.69
	Shade	9.38 Aa	10.72 Aa	10.05 a				
	Average	9.76 A	10.09 A	-				
Casein (%)	Sun	0.78 Aa	0.73 Aa	0.75 a	0.328	0.799	0.388	9.67
	Shade	0.78 Aa	0.81 Aa	0.79 a				
	Average	0.78 A	0.77 A	-				
SCC (×1000 SC per mL)	Sun	4.67 Aa	4.66 Aa	4.67 a	0.767	0.725	0.854	3.65
	Shade	4.66 Aa	4.62 Aa	4.64 a				
	Average	4.67 A	4.64 A	-				

TDE—total dry extract; DDE—degreased dry extract; SCC—somatic cell count; UNM—urea nitrogen in milk; CV—coefficient of variation. Averages followed by distinct uppercase letters in the line and lowercase letters in the column differ statistically (*p* > 0.05).

**Table 8:** Yield of fresh cheeses obtained from mixed cows submitted to different breeding environments.

Variable	Ambient	Additive		Average	*p* Value			
		Yes	No		Ambient	Additive	Ambient × Additive	CV (%)
Yield (%)	Sun	22.83 Aa	21.75 Aa	22.29 a	0.846	0.332	0.535	5.68
	Shade	22.29 Aa	22.04 Aa	22.16 a				
	Average	22.56 A	21.89 A	--				
Milk kg/cheese kg	Sun	4.40 Aa	4.61 Aa	4.50 a	0.845	0.297	0.563	5.43
	Shade	4.50 Aa	4.56 Aa	4.53 a				
	Average	4.45 A	4.59 A	-				

CV—coefficient of variation. Averages followed by distinct uppercase letters in the line and lowercase letters in the column differ statistically (*p* > 0.05).

**Table 9:** Physical–chemical attributes of fresh cheeses obtained from mixed cows submitted to different breeding environments and fed with phytogenic additives.

Variable	Ambient	Additive		Average	*p* Value			CV (%)
		Yes	No		Ambient	Additive	Ambient × Additive	
pH	Sun	6.90 Aa	6.92 Aa	6.91 a	0.745	0.410	0.745	0.53
	Shade	6.91 Aa	6.92 Aa	6.92 a				
	Average	6.90 A	6.92 A	-				
Acidity (% lactic acid)	Sun	0.090 Aa	0.093 Aa	0.091 a	0.206	0.073	0.443	3.70
	Shade	0.087 Aa	0.092 Aa	0.089 a				
	Average	0.088 A	0.092 A	-				
Humidity (%)	Sun	57.13 Aa	58.71 Aa	57.92 a	0.586	0.633	0.706	5.67
	Shade	56.81 Aa	57.00 Aa	56.90 a				
	Average	56.96	57.85	-				
Protein (%)	Sun	16.43 Aa	16.53 Aa	16.48 a	0.186	0.913	0.997	9.83
	Shade	17.68 Aa	17.78 Aa	17.73 a				
	Average	17.06 A	17.15 A	-				
Fat (%)	Sun	22.45 Aa	21.48 Aa	21.96 a	0.347	0.767	0.067	3.46
	Shade	21.99 Aa	22.72 Aa	22.35 a				
	Average	22.22 A	22.10 A	-				
TDE (%)	Sun	43.87 Aa	41.28 Aa	42.58 a	0.753	0.407	0.470	7.29
	Shade	43.19 Aa	43.00 Aa	43.09 a				
	Average	43.53 A	42.14 A	-				
FDT (%)	Sun	51.35 Aa	52.37 Aa	51.86 a	0.797	0.442	0.831	6.56
	Shade	51.42 Aa	53.21 Aa	52.32 a				
	Average	51.38 A	52.89 A					
Ash (%)	Sun	2.67 Aa	2.45 Aa	2.56 a	0.654	0.237	0.413	7.59
	Shade	2.63 Aa	2.58 Aa	2.60 a				
	Average	2.65 A	2.52 A	-				

TDE—total dry extract; FDT—fat on dry extract; CV—coefficient of variation. Averages followed by distinct uppercase letters in the line and lowercase letters in the column differ statistically (*p* > 0.05).

## Data Availability

The datasets generated during and/or analysed during the current study are available from the corresponding author upon reasonable request.
